# Relationship between testosterone levels and depressive symptoms in older men in Amirkola, Iran

**Published:** 2014

**Authors:** Farzan Kheirkhah, Seyed Reza Hosseini, Seyyedeh Fatemeh Hosseini, Nafiseh Ghasemi, Ali Bijani, Robert G. Cumming

**Affiliations:** 1 Social Determinants of Health (SDH) Research Center, Babol University of Medical Sciences, Babol, Iran.; 2 Babol University of Medical Sciences, Babol, Iran.; 3 School of Public Health, University of Sydney, New South Wales, Australia.

**Keywords:** Depressive symptoms, Testostrone, Older people

## Abstract

***Background: ***Testosterone may be an important factor causing depression in the elderly men. The purpose of this study was to determine the relationship between testosterone levels and depressive symptoms in older men in Amirkola, Iran.

***Methods:*** This cross- sectional study is a part of the Amirkola Health and Aging Project **(**AHAP**)** that involves people aged 60 and above living in Amirkola, a small town in northern Iran. The testosterone levels were measured using ELISA on morning blood samples (ngr / ml) and depressive symptoms were identified using Geriatric Depression Scale (GDS). The data were collected and analyzed.

***Results:*** Eight hundred thirty elderly men with the mean age of 70.02±7.7 years were included. On the basis of GDS criteria, 593 individuals had no depressive symptoms and 237 had at least one of these symptoms. The mean serum testosterone level in men without symptoms of depression (4.94±4.22) ngr/ml and was higher than in those with such symptoms (4.19±3.65) ngr/ml (P=0.011). Also, there was a significant inverse correlation between the testosterone levels and number of depressive symptoms (P=0.015, r=-0.084). After adjusting with age and educational levels, and living alone (OR=2.6, 95% CI: 1.17-5.82, P=0.02), testosterone levels (OR=1.67, 95% CI: 1.03-2.72, P=0.038) had the greatest impact on the development of depression.

***Conclusion: ***The results of this study showed a significant inverse relationship between serum testosterone levels and depressive symptoms in elderly men.

According to the World Health Organization (WHO), the elderly is defined as an individual over 65 years in developed countries and over 60 years in developing countries (1). Economic progress and advancement of public health has increased the elderly population in different countries. Increased life expectancy has caused the elderly population to be the fastest growing group in recent years (2). The prevalence of mental disorders, especially depression has significantly increased in old age than in other age groups. Since depression is a heavy burden, it is considered as one of the major problems of the elderly in societies (3). Medications, inheritance, life stressors, lack of social support, personality disorders, concurrent physical illness and decreased testosterone levels are among the etiological factors for depression in elderly individuals (4-7). Testosterone is the main male sex steroid hormone. The effects of this hormone on various aspects of human body and temper were described (8).

Some studies in recent years have shown that the levels of testosterone and other sex hormones and the activity of hypothalamus - pituitary - gonadal axis in depressed women were significantly lower than women without depressive symptoms (9, 10). Due to the reduced testosterone production during aging and the relationship between testosterone deficiency and depressive symptoms, probably increased prevalence of depression in old age may be attributed to testosterone deficiency (11, 12). Treatment of depression in the elderly is associated with many challenges and detection of new influential factors on geriatric depression can help find new treatments (2, 13). Many studies are being carried out on the relationship between testosterone levels and depression in the elderly (14-17). Due to the different results of these studies and the importance of depression in old age, the aim of this study was to determine the relationship between testosterone level and depressive symptoms in older people. 

## Methods

This cross - sectional study came from the "Amikola Health and Aging Project (AHAP) that was undertaken among older people 60 years of age and older in Amirkola (18). The two health centers in Amirkola have the list of all the senior citizens. Contact by phone or home visit, while providing the necessary information about the plan, the elderly were invited to participate in the study. The present study was performed on 830 men. The presence of depressive symptoms in the elderly was examined using a standard GDS (Geriatric Depression Scale) questionnaire. It contains 15 questions and each question rates one point. Ten questions indicated the presence of depression when answered positively, while the rest (question numbers 1, 5, 7, 11, 13) represented depression when answered negatively. Based on the scores, the subjects were divided into 4 groups. Zero to four: normal, five to eight: mild depression, nine to eleven: moderate depression, twelve to fifteen: severe depression. The test sensitivity was 92% and specificity was 89% (19). The Cronbach's alpha of this questionnaire was 0.81 in the elderly population of Amirkola.

Testosterone levels were measured using diametra kit made ​​in Germany by ELISA method (ngr / ml). Normal range was between 1.8 to 9. This kit only measures total testosterone levels. Fasting samples for the patients took place in the morning. The cases with history of intake of androgenic drugs in the past one month were excluded from the study. Also, the role of the other effective factors on the relationship between testosterone levels and depression such as age, level of education, living alone was adjusted by statistical methods like regression. The data were collected and analyzed by SPSS Version 18. T-test and ANOVA were used for the quantitative variables and chi-square test for the categorical variables. A p-value of 0.05 or less ​​was considered statistically significant.

## Results

Eight hundred thirty men with the mean age of 70.02±7.7 years (ranged 60-92 years) were evaluated. Majority of them were aged 60-64. Among those who participated in this study, 593 individuals had no symptoms of depression and 237 persons had depressive symptoms. In this population, 772 individuals (93%) were married and in terms of education, most of them (60.7%) were illiterate (table 1).

**Table 1:** Distribution of demographic data of older people in Amirkola (2011-12)

**Variables**	**Number**	**Percentage**
**Marital Status**		
Married	772	93.0
Widow	4	0.5
Divorced	1	0.1
Separated	1	0.1
Widower	52	6.3
Total	830	100.0
**Living alone**	26	3.1
**Education Level**		
Illiterate	504	60.7
Elementary school, reading and writing	216	26
Junior high school, High school, diplomas	68	8.2
Post Diplomas, Bachelor, Masters	10	1.2
Total	830	100

**Figure 1:**
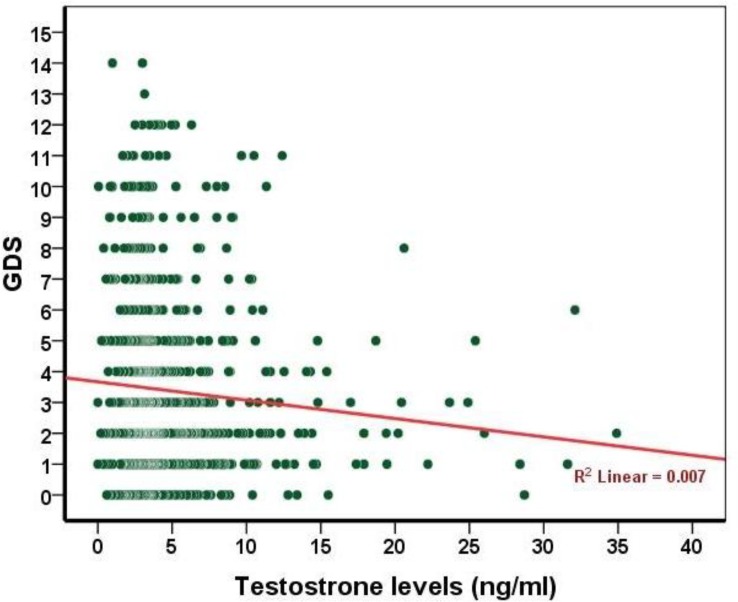
The relationship between testosterone levels and GDS score in the older people in Amirkola (2011-12)

The mean testosterone levels in patients with no symptoms of depression (4.94±4.22) were significantly greater than those with depressive symptoms (4.19±3.65) (P=0.011(. There was no significant correlation between age and GDS (r=0.051, P=0.143). There was a significant inverse relationship between testosterone levels and depressive symptoms, meaning that as testosterone levels decrease, depression scores increase (P=0.015, r= 0.084) (figure 1). The mean serum testosterone levels were lower in the individuals without depressive symptoms than those with depressive symptoms and higher GDS score, although this difference was statistically insignificant (table 2) (P=0.118). Also as the testosterone levels increase, the attitude of the elderly towards their health improved.

**Table 2:** Distribution of older people in Amirkola according to GDS scores and testosterone levels (Mean±SD) in 2011-12

**Variable**	**N**	**Testosterone levels** **(Mean±SD** **)**
**GDS Score**		
Normal Low Moderate Severe	593 173 51 13	4.94±4.22 4.25±3.94 4.11±2.98 3.74±1.33
**Self health rate**		
Very bad Bad Relatively good Good Excellent for my age	9 45 137 455 184	3.67±1.77 4.69±4.93 4.27±3.38 4.89±4.29 4.71±3.85

As shown in table 3**, **in the logistic regression model, after adjusting with other effective variables on depression such as age and educational levels, and living alone [OR=2.6,95%CI: 1.17-5.82, P=0.02], testosterone levels [OR=1.67, 95% CI: 1.03-2.72, P=0.038] had the greatest impact.

**Table 3:** Odds ratio and 95% confidence interval of variables affecting GDS score in the older people in Amirkola (2011-12)

** Variables**	**OR** *	**OR95% C.I** *		**Pvalue**
		**Upper**	**Lower**	
**Testosterone **(1.8ng/ml)	1.671	2.717	1.028	0.038
**Education level**				0.494
Illiterate	reference			
Elementary school, reading and writing	0.924	1.331	0.641	0.671
Junior high school, High school, Diplomas	0.683	1.275	0.365	0.231
Post diplomas, Bachelor, Masters	0.634	1.398	0.288	0.259
**Age group**				0.668
60-64	reference			
65-69	0.908	1.421	0.581	0.673
70-74	1.211	1.900	0.772	0.404
75-79	0.980	1.575	0.610	0.934
>=80	0.810	1.389	0.472	0.444
**Living alone**	2.603	5.817	1.165	0.020
Constant	0.397			0.000

* OR=Odds Ratio

* CI=Confidence Interval

## Discussion

As life expectancy increases, elderly population grows. The prevalence of mental disorders, especially depression significantly increased in old age than in other age groups and depression in the elderly often showed resistance to conventional treatments. Therefore, identifying the factors that influence the development and course of depression treatment is important (12). 

In the present study, the correlation between testosterone levels and depression in elderly men was evaluated. It was concluded that men with depressive symptoms had lower total testosterone levels than those without depressive symptoms. A significant inverse correlation was found between testosterone levels and depressive symptoms such that depression scores were lower with increased testosterone levels. Testosterone is one of the hormones that is secreted in response to social, sexual, physiological factors and affects the brain as a target organ thereby affecting the mood and behavior of individuals (10). So the low levels of testosterone lead to decreased energy and libido, discomfort, pain and sleep disturbances (20). The results of Health ABC STUDY that examined the relationship between testosterone levels and depression by measuring the total testosterone levels was similar to the results obtained in our study (21). In Barrett-connor et al’s study, the measurement of testosterone and depression was performed by RIA method and Beck depression scale, respectively. 

The results represent that decreased levels of free testosterone leading to increased depression and this relationship is independent of age, body weight change and physical activity. These three factors were important confounders of the relationship between sex hormones and Beck depression scale. His study consisted of middle-aged to elderly men however, in our study, this relationship has only been evaluated in the elderly (22). 

The results of Massachusetts Male Aging Study (MMAS), which included 1709 men aged 40 to 70 years showed that each unit increased in the concentration of total serum testosterone results in a 10% reduction in the risk of depression (23). In Zarroaf et al’s study showed the efficacy of testosterone in the treatment of depression and believed that testosterone can be effective as antidepressants in depressed patients. They suggested that in patients with depression, particularly in elderly men, the testosterone levels should be measured (24). They also showed that the effects of testosterone besides other therapies had been impressive in the treatment of resistant depression (25). 

The study performed by Seidman and Stuart showed that mild and chronic depressive syndromes in elderly men are associated with decreased function of the HPG axis and one of their findings was a decrease in testosterone levels (26). In addition to reduced testosterone concentration with aging, testosterone secretion is usually highest in the morning and lowest in the afternoon (27). To reduce this impact in our study, morning fasting blood samples were used to measure testosterone levels in all samples. Osvaldo in his cross-sectional study involving 3987 men aged 71 to 80 years concluded that there was a relationship between depression and low testosterone levels and testosterone can be useful for the treatment of depression in hypo-gonadal old men (15). Rieter performed a study on 240 middle-aged depressed men. He once treated them with a low dose of testosterone (800-1800 mg) that caused a modest decline in their depression scores. Then, he again treated them with high doses of 2000-2400 mg. It reduced their depression (28). Davis et al. in their study used the saliva samples to measure testosterone levels. They compared 11 depressed men with 10 non-depressed men. 

They observed no difference in testosterone levels in the two groups, but in depression group alone, free testosterone levels had a significantly negative correlation with severity of depression (29). In another study, a group was treated with testosterone and other group with placebo, the difference was insignificant (30). Roose et al. performed their study on 30 men with major depressive disorder (mean age 52±8 years) and the total testosterone levels were lower than normal. After treating one group with testosterone and another group with placebo, they observed a similar response (31). 

This study has some limitations. First, it was a cross sectional study so the explanation of causality in these studies is difficult. Second, this study was based on the evaluation of only a single hormone which was the total testosterone levels measured only in the morning. If the measurement of several hormones could be performed at different time intervals, this study would be more accurate. Third, in this study we did not compare the two sexes and only the elderly men were examined. 

The use of a large sample size and being a population-based study where in the participants had not taken androgenic drugs are the strengths of this study. According to the findings of this study and other studies, to confirm the role of testosterone in reducing depression and its therapeutic effect, further evaluations via prospective studies, laboratory experiments and eventually an experimental study on the patients are needed.

## Notes

**Funding: **This study was supported by a grant from the Vice-Chancellery of Research and Technology of Babol University of Medical Sciences (grant no. 892917).
